# The characterization of a hospitalized population at the pediatric emergency service of Mother and Child Hospital, Marrakech, Morocco

**DOI:** 10.11604/pamj.2022.43.138.29471

**Published:** 2022-11-14

**Authors:** Siham Jbari, Widad Lahmini, Mohamed Eddabbah, Samia Boussaa, Mounir Bourrous

**Affiliations:** 1 Infectious Disease Research Laboratory, Faculty of Medicine and Pharmacy, Cadi Ayyad University Marrakech, Morocco,; 2 Childhood Health and Development Research Laboratory, Faculty of Medicine and Pharmacy, Cadi Ayyad University Marrakech, Morocco,; 3 Higher Institute of Nursing and Health Techniques (ISPITS), Marrakech, Morocco,; 4 Higher Institute of Nursing and Health Techniques (ISPITS), Rabat, Morocco

**Keywords:** Pediatric emergency services, pediatric pathologies, epidemiological profile

## Abstract

**Introduction:**

the pediatric emergency department is the first contact between the population and the hospital. Consequently, its dysfunction influences the quality of the general health care system. However, any successful policy must first be based on convincing results hence the need to better explore this service, diagnose the various dysfunctions, and survey disease trends to identify the needs of the local population. In this perspective, we propose to describe the epidemiological profile of children hospitalized at the emergency service of the Mother-Child hospital, University Hospital Centre Marrakech, and establish the prevalence table for childhood pathologies.

**Methods:**

a retrospective study was carried out in pediatric emergency services for 1658 hospitalized patients between March 2015 and December 2018. The collected data concerns mainly the socio-demographic, clinical profile, evolution status, mode of admission, and medical history.

**Results:**

the characterization of the studied population by sex and age showed a predominance of male with a sex ratio of 1.36, infants with 625 patients. Concerning the final diagnosis, the most frequent pathologies affected the respiratory system in 28% of cases, then the digestive system (11.3%), while infectious pathologies represented 10.7% of admissions. The death rate in the emergency department was 7.4%. Multivariate analysis of the data showed a statistically significant relationship between the final diagnosis (16 diseases by a system according to The International Statistical Classification of Diseases and Related Health Problems 10th Revision (ICD-10)) and age, season, and weight. Thus, for the association between the digestive system diseases and weight (aOR=1.052, 95% CI= 1.019-1.086, p=0.02). While for the skin and subcutaneous tissue diseases and the autumn season (aOR=11.37, 95% CI= 1.272-101.777, p=0.03) and age has a negative significance for most diseases.

**Conclusion:**

the epidemiological profile study will allow knowledge of patient´s pathologies typology for a well-supported and better definition of needs.

## Introduction

Infant mortality decreased from 11.9 million deaths in 1990 [1] to 5.6 million in 2016 [2]. Despite this progress, child mortality remains high worldwide [3]. According to the WHO (2012), 60 million children will die before the age of 5 years by 2030, 80% of whom are children in sub-Saharan Africa and South Asia [4]. The fight against child mortality is based on the quality of care provided in pediatric emergencies [5]. By definition, pediatric emergencies are all morbid conditions that threaten a child´s life in a more or less short period and require rapid and adequate care [6]. This definition is not always respected because of the pediatric emergency department (PED) heterogeneity and the different perceptions of healthcare by staff and parents [5,7]. As a result, the PED suffers from overcrowding, which has become a universal and growing problem. In France, for example, between 1990 and 2001, there was a 64% increase in the number of visits to emergency departments in public institutions, an annual increase of 6.4% [6]. In Switzerland, authors report an increase in the number of urgent pediatric consultations at least 16% between 1990 and 1999 [8]. In sub-Saharan Africa (Congo), pediatric medical emergencies report 18.54% of admissions [6].

Morocco has not escaped this phenomenon with an inflation of more than 20% in the rate of pediatric emergencies number at the Ibn Sina CHU (Rabat city) between 2014 and 2015 [9]. Whereas at the Ibn Rochd CHU (Casablanca city), the visits number to the pediatric emergency department increased from 40,786 in 2007 to 64,507 in 2016 [10]. Under these conditions, the PED with its three levels: reception, sorting, and hospitalization [10] could be negatively affected. Several studies [11,12] have looked at the various dysfunctions in pediatric emergencies in developed countries, particularly in terms of reception and sorting (false emergencies, user satisfaction, and mortality and morbidity in emergencies). On the other hand, very little research has focused on the situation of hospitalization in the PED in developing countries [12,13]. The present study is part of the regionalization policy adopted by Morocco and its commitments with the WHO, which aims to improve access to care and improve the tertiary level [2]. It aims to describe the epidemiological profile of children hospitalized in the pediatric emergency departments of Mohammed VI CHU of Marrakech-Safi region and to evaluate the presentation models and offered services. These convincing results will help to inform health policies and improve pediatric emergency practice at the national level.

## Methods

### Type and setting of the study

**Study design**: a cross-sectional study described the epidemiological profile of children in the PEDs of the Mother and Child Hospital, Mohammed VI (CHU) (Marrakech City) between March 2015 and December 2018. Also, we try to determine the association between the final diagnosis and different factors in the study.

**Study setting and population**: this retrospective study was conducted in June 2019 based on hospitalized patients records in the PEDs of the Mother and Child Hospital, Mohammed VI (CHU) (Marrakech City) between March 2015 and December 2018. The Mohammed VI CHU (Marrakech city) is a public level three hospital composed of four hospitals and two centers, with a capacity of 1548 beds [14]. It essentially covers the region of Marrakech-Safi, but also the territorial regions of central and southern Morocco. The Mother and Child Hospital are one of the four hospitals of the CHU which has a gynecological-obstetrical and pediatric vocation, with a bed capacity of 247 beds. Its pediatric center has more than six departments [15] including the pediatric emergency service (PED) which is considered the only specialist department in the southern region of Morocco.

**Study sampling**: after the elimination of incomplete patient records, a total of 1658 records were retained for this study, concerning children aged 0 to 16 years, hospitalized in the emergency department of the Mother and Child Hospital for a medical or surgical pathology, between 2015 and 2018.

**Study variables**: the targeted variables mainly concern: (a) the patient´s socio-demographic profile, (b) the reason for hospitalization and history, (c) the mode of transfer and origin of referral, and (d) the nature of care and progress. Then, the association between the final diagnosis (dependent factors) and different independent factors were analysed in the study. This dependent variable (system by pathology) reflects the final diagnosis according to the ICD-10 classification of the WHO.

**Data resource and measurement**: data collection: we use a pre-established survey form, on admissions to the PED hospitalization unit from March 2015 to December 2018 and extracted data from patients´ records. The data were analyzed using SPSS (Version 18). Variables with missing data were discarded from the analysis. We started the statistical tests between our dependent variable (the final diagnosis) and the independent factors. Moreover, we first checked the robustness of our model with a P=0,00 which reflects that our model is well-adjusted. For Frequencies and percentages were computed as descriptive statistics. We followed a backward stepwise approach to select variables to build the best model. Thus, the X^2^ test (Chi-square two) was used to compare proportions with a significance level of 0.05 and the Cramer´s V test was used to study correlations. Also, we measured the association between independent significance in Chi square and dependent variables by using a regression multinomial to find the association and predict the risk of diseases. Regarding the Multivariate analysis, all factors that were significant in univariate analysis were put in multivariate analysis. Each identified factor was then presented with its adjusted odds ratio (ORa) and its IC95% In all tests, the p threshold was set at 5% (p < 0.05).

**Ethical considerations**: the study was carried out after getting authorization from the local and regional health services (Reference N°2692/21). The confidentiality of data and privacy of all participants was assured. The study was conducted in accordance with the Declaration of Helsinki.

## Results

After elimination of incomplete patient records, a total of 1658 records were retained for this study, concerning children aged 0 to 16 years, hospitalized in the emergency department of the Mother and Child Hospital for a medical or surgical pathology, between 2015 and 2018.

**Socio-demographic characteristics of patients**: for our sample (Table 1), the extremes of age in days are from (Min=0) to 5840 (Max=16 years), while the median is 120 days and the mode equals 365 days (1 year). According to WHO standards (49), we divided patients into age groups: Newborn, Infant, Toddler, Large Child, and Adolescent (Table 1). Thus, we noted the predominance of the infant category with 625 (38%) patients, whereas adolescents constitute the least represented age group with 130 patients (7.8%). The sex ratio is 1.36 which means 957 boys against 701 girls. According to the origin, 88.8% of patients hospitalized at the PED between 2015 and 2018 are from the Marrakech- Safi region, followed by the Drâa-Tafilalt region with 4.30% (Table 1).

**Table 1:** distribution of the study population by age, gender, and region

Variable	Frequency	Percentage(%)
**Age**		
New born: 0 to 28 days	545	33
infant: 1 month to 2 years	625	38
small child: 2 to 5 years	168	10
Big child: 5 to 10 years	190	11.2
Teenager: 10 to 16 years	130	7.8
**Sex**		
Male	957	57.8
Female	701	42.2
**Region (cities)**		
Marrakech-Safi	1375	88.8
Drâa-Tafilalt	67	4.30
Béni Mellal-Kénifra	60	3.87
Souss-Massa	25	1.61
Laâyoune-Sakia EL Hamra	07	0.45
Casablanca-Settat	06	0.40
Tanger-Tétouan-El Hoceima	03	0.19
Guelmim-Oued Noun	03	0.19
Rabat-Salé-Kenitra	02	0.13
Dakhlaa-Oued Ed Dahab	01	0.06

**Conditions of referral, transfer, and hospitalization**: according to Table 2, self-referral (by parents or a relative) is the most dominant 75%, with low representation of other referral institutions. For the means of transportation to the emergency departments, it is mainly by the patients´ means (85.70%). We have considered three levels of the hospitalization duration: 24 hours, 48 hours, and more than 48 hours (Table 2). The collected data shows duration of hospitalization between 1 day and 28 days, with an average of 4 days and the mode is 2 days. We note that more than half of the hospitalized children (64.75%) spent more than 48 hours in the hospital (Table 2).

**Table 2:** distribution of patients according to referral, transfer, and hospitalization conditions

Variable	Frequency	Percentage(%)
**Reference origin**		
Parents or a relative	1248	75.27
A pediatrician	76	4.58
Health center	55	3.32
General practitioner	147	8.87
Childbirth home	36	2.17
Provincial Hospital	75	4.53
others	21	1.26
**Means of transport**		
Ambulance	222	13.40
Personal means	1424	85.70
SAMU	15	0.90
**Type of emergency**		
Medical	1494	90.10
Surgical	164	9.90
**Background of the child**		
None	942	56.80
Medical	624	37.70
Chirurgical	62	3.70
Medical + Surgical	30	1.80
**Duration of hospitalization**		
24h	123	12.80
48h	216	22.45
>48h	623	64.75
**Treatments received before the consultation**		
Aucun	1434	86.50
Medical	181	10.91
Traditional	43	2.59

SAMU: The Emergency Medical Assistance Service (*French: Service d´Aide Médicale Urgente*)

**Reasons for consultations and background**: the most dominant is medical emergencies with 90.10% (Table 2). The most represented consultation reason has a respiratory origin (25%), then infectious origins, represented by fever, diarrhea, vomiting (5%), exposure to a local traditional healer for children (FERRAGA) (3%) and foreign body´s ingestion (2%). For the patient medical history, 56.80% with no previous medical or surgical history, while 37.7% presented a medical history and 5.5% presented a surgical history (Table 2). According to the results, the majority of patients (86.5%) came to the emergency room without prior treatment (Table 2).

Final diagnosis and evolution: the main diagnoses are respiratory (17.8%), digestive (5.4%), infectious (4.10%), urinary (3.60%), neuromeningeal (2.20%) and pathologies related to prematurity (2.20%), followed by ingestion of foreign bodies (2.10%). The final diagnoses according to the International Classification of Diseases (Table 3) are, in descending order, diseases of the respiratory system (28%), diseases of the digestive system (11.30%), certain infectious and parasitic diseases (10.7%), and diseases of the genitourinary system (9.8%). While the evolution of these patients is favourable with a discharge rate of 67.60%, compared with an emergency room mortality rate of 7.40%. Table 4 presents the results of the statistical correlations between the final diagnosis and different factors recorded. According to the X^2^ test, we noted the absence of a relationship between the final diagnosis with the origin (urban/rural) and the gender of the patients. On the other hand, we concluded a statistically significant relationship (P<0.05) between the final diagnosis and age and the treatment received before consultation (V de Cramer between 0.20 and 0.40); then with the region and medical history of the child (V de Cramer between 0.10 and 0.20) and finally with the reason for hospitalization (V de Cramer between 0.80 and 1). We have also spread the duration of the study over the four seasons to deepen the analysis. Table 4 presents the results of the univariate analysis of the final pathologies and the different variables according to the four seasons: season, age group, and treatment received before the consultation. We found a relationship with the seasons of the year, age group, and self-medication (P <0.05).

**Table 3:** the final diagnoses according to the international classification of pathologies (WHO, 2015) and the evolution of children hospitalised at the pediatric emergency department in Marrakech city

Diagnosis	Number (N)	Percentage(%)
Injury, poisoning and certain other consequences of external causes	130	7.9
Disease of the circulatory system	71	4.30
Disease of the digestive system	187	11.30
Diseases of the nervous system and sense organs	91	5.50
Diseases of the respiratory system	463	28
Diseases of the genitourinary system	163	9.8
Multi-sided attack	131	7.90
Diseases of the skin and subcutaneous tissue	11	0.70
Endocrine, nutritional and metabolic diseases	18	1.10
State of shock (external causes of morbidity and mortality)	14	0.80
Disease of the blood and blood forming organs and certain disorders	45	2.70
Certain infectious and parasitic diseases	177	10.70
Diseases of the eye and its adnexa	37	2.20
Premature(certain conditions originating in the perinatal period)	87	5.20
Diseases of the musculoskeletal system and connective tissue	32	1.90
**Evolution**	**N**	**%**
Death	122	7.40
possible hospital discharge	1121	67.60
Leaving against medical advice	54	3.3
Transfer	26	16
Others	335	20.10

**Table 4:** statistical association of the final diagnosis and the different variables of the study

Variable	N	χ^2^ value	ddl	Value p	P<0,05	V value of cramer
**Origin**	1658	867,309^a^	867	0.491		0.175
**Sex**	1658	8.651^a^	117	0.951		0,072
**Age**	1645	2659.284^a^	8816	0.000	*	0.308
**Region**	1549	210.011^a^	1153	0.002	*	0.123
**Reason for hospitalization**	1658	22971.270^a^	114552	0.000	*	0.903
**Treatment received before consultation**	1658	467.081^a^	551	0.000	*	0.306
**child medical history**	1658	261.518^a^	668	0.000	*	0.199

* Means p value is significant

Regarding the Multivariate analysis, all factors that were significant in univariate analysis were put in multivariate analysis and only three (season, weight, age range) were found to be statistically associated with the occurrence of the final diagnosis. In (Table 5, Table 5 (suite), Table 5 (suite 1)) we note that the B value has an increasing or decreasing influence on the final diagnosis if it is negative or positive. For the weight in reference to infectious diseases, the association is negative for diseases of the respiratory system and prematurity-newborn with a risk rate ranging from 0.96% for respiratory diseases. For the rest, we can qualify as positive because when the weight increases the risk of these pathologies increases. Moreover, the seasons we have a positive association varying by pathology between 11 times (diseases of the skin and subcutaneous tissue in Autumn) with the presence of a 1% risk during the three seasons (autumn, winter, and spring) for the pathologies of system respiratory, digestive and genitourinary compared to the reference infectious diseases. Similarly, the age groups varying from 1 day to 10 years, the B value is negative. Ergo, age contributes in the opposite direction, so that as age increases, the risk decreases, with a maximum rate of 0.61% (multivisceral pathologies) for ages between 5 and 10 years concerning multivisceral damage.

**Table 5:** multivariate logistic regression for the final diagnosis of the different variables of the study

Final diagnosis	B	P -value	aOR	95% C.I	
				Lower	Upper
**Accident and Injury**					
**weight by kg**	0.104	0.000	1.110	1.058	1.165
[season =1]	1.151	0.003	3.161	1.498	6.670
**Diseases of the respiratory system**					
**weight by kg**	-0.036	0.032	0.965	0.934	0.997
[age range=1.00]	-4.844	0.002	0.008	0.000	0.174
[age range=2.00]	-3.808	0.010	0.022	0.001	0.409
[age range=3.00]	-4.177	0.002	0.015	0.001	0.218
[season =1]	0.602	0.030	1.826	1.059	3.150
[season =2]	1.741	0.000	5.704	3.435	9.470
[season =3]	0.561	0.017	1.753	1.106	2.777
**Diseases of the blood and blood forming organs**					
**weight by kg**	0.051	0.041	1.052	1.002	1.104
[age range=2.00]	-4.641	.027	.010	.000	.589
**Diseases of the digestive system**					
**weight by kg**	0.051	0.002	1.052	1.019	1.086
[age range=2.00]	-5.163	0.001	0.006	0.000	0.114
[age range=3.00]	-5.548	0.000	0.004	0.000	0.062
[age range=4.00]	-3.057	0.012	0.047	0.004	0.509
[season =1]	0.822	0.008	2.276	1.244	4.165
[season =2]	0.622	0.049	1.862	1.001	3.462
[season =3]	0.578	0.032	1.783	1.051	3.024

**Age range**: 1(New Born: 0 To 28 days), 2(infant: 1 month to 2 years), 3(small child: more than 2 to 5 years),4(big child: more than5 to 10 years) **Saison**: (1) Autumn, (2) winter, (3) spring, (4) summer **aOR**: odd of ratio **CI**: confidence interval

**Table 5 (suite):** multivariate logistic regression for the final diagnosis of the different variables of the study

Final diagnosis	B	P -value	aOR	95% C.I	Final diagnosis
				Lower	Upper
Diseases of the musculoskeletal system and connective tissue					
[season =1]	1.274	0.017	3.576	1.260	10.153
Diseases of genitourinary system					
[season =1]	0.874	0.006	2.396	1.285	4.466
[season =2]	0.651	0.047	1.917	1.009	3.641
[season =3]	0.552	0.050	1.737	1.000	3.015
Poisoning					
[season =2]	1.516	0.000	4.552	1.955	10.599
Diseases of the skin and subcutaneous tissue					
weight by kg	0.142	0.000	1.153	1.078	1.233
[season =1]	2.432	0.030	11.379	1.272	101.777
Ophtalmolo diseases of the eye and adnexa					
weight by kg	0.095	0.000	1.100	1.047	1.155
[season =1]	1.410	0.019	4.097	1.262	13.294
[season =2]	1.921	0.001	6.828	2.267	20.558
Diseases of the circulatory system					
[season =2]	0.928	0.019	2.529	1.168	5.473

**Age range**: 1(New Born: 0 To 28 days), 2(infant: 1 month to 2 years), 3(small child: more than 2 to 5 years),4(big child: more than5 to 10 years) **Saison**: (1) Autumn, (2) winter, (3) spring, (4) summer **aOR**: odd of ratio **CI**: confidence interval

**Table 5 (suite 1):** multivariate logistic regression for the final diagnosis of the different variables of the study

Final diagnosis	B	P -value	aOR	95% C.I	Final diagnosis
				Lower	Upper
**prematurity-newborn (Certain conditions originating in the perinatal period)**					
**weight by kg**	-1.333	0.000	0.264	0.180	0.387
[season =1]	0.742	0.039	2.101	1.037	4.256
[age range=2.00]	-4.989	0.008	0.007	0.000	0.267
[age range=3.00]	-4.533	0.007	0.011	0.000	0.293
**multivisceral damage**					
[age range=1.00]	-4.392	0.013	0.012	0.000	0.400
[age range=3.00]	-2.909	0.047	0.055	.0003	0.968
[age range=4.00]	-2.797	0.037	0.061	0.004	0.839
[season =1]	1.122	0.002	3.072	1.489	6.341
[season =2]	1.797	0.000	6.031	3.072	11.842

**Age range**: 1(New Born: 0 To 28 days), 2(infant: 1 month to 2 years), 3(small child: more than 2 to 5 years),4(big child: more than5 to 10 years) **Saison**: (1) Autumn, (2) winter, (3) spring, (4) summer **aOR**: odd of ratio **CI**: confidence interval

## Discussion

The study requiring hospitalization pathologies (at the PED) has highlighted the specific nature of pediatric emergencies. For gender, there is a slight predominance of the male sex with a sex ratio of 1.36. This corresponds to the majority of studies carried out on pediatric emergencies in Maghreb [16-18], Africa [5,6,19], and developed countries [20,21]. This male predominance can be explained by biological differences indicating a vulnerability of gender origin for the male sex more than the female sex [12,22] and by the general demographic distribution of the population in the Morocco kingdom, where there has been a slight increase in the male sex to the female sex for the under 15 age group [23]. Other studies put forward the concept of culture as a factor influencing the decision of parents to come to the emergency departments, favouring the male sex for healthcare [12,22]. For the age group, we have a predominance of infants (38%) and newborns (33%). This is consistent with studies that suggest that infants are the most common age group attending emergency departments [12,24,25]. Infants are said to be more fragile, particularly due to the immaturity of their immune system and the presence of certain congenital diseases at this age [26]. Our results highlighted a significant positive correlation between the final diagnosis and the age of the patients (Chi-square two<0.05). We can deduce that age and final diagnosis are related in such a way that frequent pathologies can be predicted according to the dominant age.

The dominance of infants and newborns (71%) in the PED implies the need for the creation of a specific neonatology unit to strengthen the existing unit and ensure healthcare to this category according to standards [27,28]. The emergency department is an environment which is not suitable for the hospitalization of premature babies, as any failure in their care can lead to risks of mortality and morbidity [11]. Hence to take care of this population, is a major challenge for the Moroccan healthcare system, The study highlighted the predominance of self-referencing (75%), as noted by other authors [19,29]. This result can be explained by the tendency of parents, when faced with a sick child, to address the PED directly [15]. This tendency demonstrates the non-respect of the health pyramid by the population; and would also explain the high number of false emergencies that clog the PEDs [30,31]. This phenomenon can only negatively affect the performance of the PEDs [32]. Similarly, the vast majority of patients make only marginal use (less than 5%) of urgent medical transport [24]. In the present study, 85.70% of the parents used their means of transport to access the PED, raising the problem of access to medical transport (cost and/or availability) as the study by LY *et al*. [32].

The average duration of hospitalization is 4 days. In the Democratic Republic of the Congo, 50% is the rate recorded for the length of hospitalization of children with an emergency sign of fewer than 4 days [7]. In France, 72% of patients stayed more than 4 days in the emergency department [26]. According to the standards, the role of the Short-Term Hospitalization Unit (STHU) in the emergency department is to make a rapid diagnosis, to start urgent treatment, and to discharge or transfer the patient within 24 to 48 hours at the most [10,33]. In our study, we note that the STHU is being transformed into a conventional hospitalization environment, which represents a major dysfunction that weighs down the activity of the department and the staff. All the more so as the number of patients hospitalized per day most often exceeds the capacity of the department [10]. Hence the interest in regulating the stay time in the emergency departments in Morocco. According to Enyuma [15], children who had received several previous treatments had a 59% to 74% chance of staying more than 72 hours at the PED level. In our context, the majority of patients (86.5%) come to the emergency room without prior treatment. Therefore, self-medication does not explain the high length of hospitalization (>48h). As regards the reasons for consultation, a total of 587 pathologies have been identified. The most frequent are diseases of the respiratory system (28%), diseases of the digestive system (11.30%), certain infectious and parasitic diseases (10.7%), and diseases of the genitourinary system (9.8%). These results agree with the literature which states that the reasons for pediatric consultations, in general, are of respiratory origin, followed by digestive pathologies characterized by gastroenteritis and then infections which differ according to country and continent [12,32,34]. In Mali, malaria and meningitis were among the main pathologies recorded in pediatric emergencies [28].

In the present work, we have noticed that the distribution of the pathologies encountered has a seasonal character. This relationship is confirmed statistically (P<0.05), also, the regression multinomial showed a high risk of association between different diseases and seasons, especially autumn and winter. Cold and hot seasons influence the respiratory and digestive pathologies of children by climate change and promiscuity [32,35]. These variations can cause real problems in the PED performances, with a risk of saturation during certain periods. Awareness of the seasonal variation in the incidence of common pediatric emergencies would allow the planning of preventive and organizational actions adapted to each season. Vital and functional prognosis remains the major concern in care end support. In the present study, the mortality rate of children reached 7.4% and this rate could be higher given the number of patients discharged against medical advice (3.3%). A study on mortality in the same department during the period 2012-2016 showed a mortality of 3.63% with a predominance of infant and child mortality (93.3%) [36], while a study in Nigeria showed a mortality of 11.6% in pediatric emergencies during the period 2015-2017 [37]. Infant mortality varies between countries and remains very low in high-income countries [38]. For example, Maniktala *et al*. [39] recorded a rate of 1.7 deaths per 10,000 admissions in the United States and Lopez *et al*. [40] noted a rate of 1.5 per 10,000 admissions in Spain. As a limitation, we cannot establish a causal association between dependent and independent variables in the present study. More investigations are needed to confirm the direction and the power of these associations and consequently generate the results.

## Conclusion

Despite its recent establishment, the PED in Marrakech city provides an important service for children at a regional and national level. Nevertheless, infant mortality and hospitalization remain concerns and problems to be addressed. All the bodies involved in the patient circuit should look into the subject to improve patient care and reduce the morbidity and mortality linked to the dysfunctions of this circuit. Our results underline, on the one hand, the predominance of respiratory, digestive, and infectious pathologies as a major source of hospitalization and, on the other hand, the distribution of these pathologies according to the seasons and the age of the child. Consequently, it is essential to prepare plans to respond to seasonal peaks in PED influxes to improve care. A better definition of the population´s needs would make it possible to target training actions for healthcare teams and optimize regional organization and patient transfers.

### What is known about this topic

- Despite this progress, child mortality remains high worldwide;
- The fight against child mortality is based on the quality of care provided in pediatric emergencies.

### What this study adds

- There are various studies on dysfunctions in pediatrics in terms of reception and triage, but very little research like ours has focused on the situation of hospitalization in developing countries;
- It is essential to prepare plans to respond to seasonal peaks in PED influxes to improve care;
- The definition of the needs for the population in the region of Marrakech-Safi will allow to satisfy the needs of the population and to improve the output.
